# Impact of FecB Mutation on Ovarian DNA Methylome in Small-Tail Han Sheep

**DOI:** 10.3390/genes14010203

**Published:** 2023-01-12

**Authors:** Lingli Xie, Xiangyang Miao, Qingmiao Luo, Huijing Zhao, Xiaoyu Qin

**Affiliations:** Institute of Animal Sciences, Chinese Academy of Agricultural Sciences, Beijing 100193, China

**Keywords:** sheep, epigenetics, ovary, methylation, fecundity

## Abstract

Booroola fecundity (FecB) gene, a mutant of bone morphogenetic protein 1B (BMPR-1B) that was discovered in Booroola Merino, was the first prolificacy gene identified in sheep related to increased ovulation rate and litter size. The mechanism of FecB impact on reproduction is unclear. Methods: In this study, adult Han ewes with homozygous FecB(B)/FecB(B) mutations (Han BB group) and ewes with FecB(+)/FecB(+) wildtype (Han ++ group) were selected. Methylated DNA immunoprecipitation and high-throughput sequencing (MeDIP-seq) was used to identify differences in methylated genes in ovary tissue. Results: We examined differences in DNA methylation patterns between HanBB and Han ++ sheep. In both sheep, methylated reads were mainly distributed at the gene body regions, CpG islands and introns. The differentially methylated genes were enriched in neurotrophy in signaling pathway, Gonadotropin Releasing Hormone (GnRH) signaling pathway, Wnt signaling pathway, oocyte meiosis, vascular endothelial growth factor (VEGF) signaling pathway, etc. Differentially-methylated genes were co-analyzed with differentially-expressed mRNAs. Several genes which could be associated with female reproduction were identified, such as FOXP3 (forkhead box P3), TMEFF2 (Transmembrane Protein with EGF Like and Two Follistatin Like Domains 2) and ADAT2 (Adenosine Deaminase TRNA Specific 2). Conclusions: We constructed a MeDIP-seq based methylomic study to investigate the ovarian DNA methylation differences between Small-Tail Han sheep with homozygous FecB mutant and wildtype, and successfully identified FecB gene-associated differentially-methylated genes. This study has provided information with which to understand the mechanisms of FecB gene-induced hyperprolificacy in sheep.

## 1. Introduction

Sheep rearing is an important component of livestock husbandry, providing both mutton and wool for modern society. The increased market demands in emerging countries—China, for example—require a greater sheep supply. Ewe prolificacy is one of the main limitations to increasing sheep production. Many factors affect sheep prolificacy, such as the length of estrus period, ovulation rate and litter size. These depend on hormone regulation and breed characteristics [1,2]. In recent decades, more and more studies have shown that genetic variations were responsible for sheep prolificacy. A series of genes was identified as prolificacy genes, including BMPR-1B (FecB), Bone morphogenetic protein 15 (BMP15) and Growth differentiation factor 9 (GDF9), etc [3]. FecB was the first fecundity gene identified, 30 years ago. Piper, Bindon and Davis found that an autosomal mutant in Booroola Merino could lead to increases of the ovulation rate and litter size [4]. Some studies demonstrated that a mutant at the bone morphogenetic protein 1B receptor (BMPR-1B, also known as ALK-6), located on chromosome 6q23-31, contributed to the FecB phenotype. This was consistent with the expression of BMPR-1B in oocytes and granulosa cells [5,6]. In addition to improving productivity efficiency in ewes, FecB gene can lower body weight and average daily gain of lambs [7,8]. The FecB mutant has been identified in numbers of prolific sheep breeds, like Small-Tail Han sheep [9].

Small-Tail Han sheep are widely bred in China, and are known for their year-round estrus and hyperprolificacy. The homozygous mutant (B/B), heterozygous mutant (B/+) and wildtype (+/+) of FecB gene have been detected in Small-Tail Han sheep [10]. The FecB mutant has significant effects on litter size in Small-Tail Han sheep [11]. The Small-Tail Han sheep with homozygous or heterozygous genotypes of FecB had 1.40 and 1.11 more lambs than wildtype, respectively [12,13]. It was found that the prolificacy effects of FecB on Han ewes were associated with expression changes of reproduction-related genes, such as follicle-stimulating hormone receptor, luteinizing hormone receptor, estrogen receptor α and progestin receptor [14]. To further study the effects of FecB gene on ovarian gene expression in Small-Tail Han ewes, we conducted transcriptomic and proteomic studies to compare gene expression differences between FecB homozygous genotype (BB) and wildtype (++) Han sheep at both mRNA and protein levels. We identified some differentially-expressed genes between Han BB and Han ++ sheep, mainly enriched in biological functions, such as ribosome assembly and mitochondrial oxidation [12,13]. However, the mechanism of the FecB mutant leading to differences of gene expression in Small-Tail Han sheep remains unclear; epigenetic mechanisms could be involved. Epigenetics refers to heritable factors that regulate gene expression without the change of DNA nucleotide sequence. Typical epigenetic mechanisms include non-coding RNAs, chromatin variation, DNA methylation and histone modifications [13,15,16,17,18]. Our previous study revealed the roles of miRNAs in gene transcription and translation modifications between Han BB and Han ++ sheep [13]. We identified methylated genes encoding mRNAs and lncRNAs that corresponded to sheep proliferation. DNA methylation is another typical epigenetic gene regulation method. It is generally related to gene transcription repression, affecting chromatin organization and RNA polymerase II binding [19]. We demonstrated the differences in ovarian genomic DNA methylation between highly prolific Small-Tail Han sheep with FecB mutant and relatively less prolific Dorset sheep without FecB phenotype [20]. Our results indicated that DNA methylation was involved in the differentially-expressed ovarian genes, leading prolificacy diversity. Differences in gene expression levels between Han BB and Han ++ sheep were identified. However, it was unclear whether the DNA methylation levels of the ovarian genomes differed between the two breeds. To answer this question, we applied methylated DNA immunoprecipitation, combined with high-throughput sequencing (MeDIP-seq), to study DNA methylation differences between Han sheep, with or without FecB genotypes. Surprisingly, this single point mutation in BMPR-1B was associated with genomic variation of DNA methylation levels.

## 2. Methods

### 2.1. Experimental Animals

In this study, 108 Han ewes were selected from a fine nucleus herd, bred by the Ao-Te sheep breeding farm in Qingdao, Shandong, China. Among these ewes, five adult Han ewes with the genotype BB and 5 adult Han ewes with the genotype ++ were separated into two groups. The adult Han ewes with the homozygous FecB(B)/FecB(B) mutation (Han BB group) and ewes with FecB(+)/FecB(+) wildtype (Han ++ group) were kept under same conditions of water and food ad libitum. All ewes in the experiment were treated to synchronize estrus [13,21].

### 2.2. The Preparation of Sample

Blood sampling was used to identify the FecB mutation in the BMPR1B gene. Then, 24 h after detection of spontaneous estrus, 3 HanBB ewes and 3 Han ++ ewes were euthanized following deep anesthesia with tiletamine/zolazepam (Zoletil 50 Vet, Virbac, Carros, France) (tiletamine 50° mg/mL and zolazepam 50° mg/mL), at a dose of 0.1 mg/kg of body weight, administered by intramuscular injection. Entire ovaries were excised to obtain better ovulation points on the surfaces of ovaries. All samples were immediately snap-frozen in liquid nitrogen and stored at −70 °C for the extraction of genome DNA and ovarian RNA.

### 2.3. MeDIP-seq Analysis

Genomic DNA was extracted from ovaries of three small-tailed ewes, with or without FecB gene mutation (Han BB and Han +/+, respectively), and mixed in equal quantities. The mixture of genomic DNA was sonicated to obtain fragments in the 100–500 bp range and dAs was added to the 3′ end of the DNA fragment. Then, an adapter was connected to both ends of each fragment with the paired ends DNA using a sample preparation kit (Illumina, Shenzhen, China) according to the manufacturer’s protocol. Double-stranded DNA was denatured to single-stranded. DNA fragments containing the methylated cytosine region were immunoprecipitated using monoclonal antibody (Calbiochem, San Diego, CA, USA) DNA anti-5-methylcytosine mice with magnetic methylation and immunoprecipitation kit (Diagenode, Denville, NJ, USA). The precipitated DNA fragments were amplified by PCR and separated by agarose gel electrophoresis using gel extraction kit (28,706; Qiagen, Hilden, Germania). The fragments of 200–300 bp were cut and extracted. DNA fragments were quantified using a Agilent2100 analyzer. The quantified libraries were sequenced in an Illumina HiSeq 2000 at the BGI (Shenzhen, China).

### 2.4. MeDIP-seq Sequence Alignments and Data Analysis

Quality control of raw data from sequencing was performed using FastQC (this is sequence alignment and data analysis, referring to the original method, followed by tagging) (http://www.bioinformatics.babraham.ac.uk/projects/fastqc/, accessed on 10 May 2022). The reads containing adapters, containing over 10% of N nucleotides or containing over 50% of contained nucleotides which had quality value Q < 21 were removed from the raw data, and all remaining reads were saved as clean reads in FASTQ files. The clean data were aligned to the sheep reference genome (Ovis_aries, ftp://ftp.ncbi.nih.gov/genomes/Ovis_aries, accessed on 10 May 2022) using SOAPaligner v2.21 (http://soap.genomics.org.cn/, accessed on 10 May 2022) with no more than 2 bp mismatches. The reads distribution in sheep chromosomes and different genome components were analyzed, including upstream2k, 5′-untranslated region (UTR), 3′-UTR, CDS, introns, CpG islands (CGIs), downstream2k and repeats, as well as the genome coverage of the CG, CHG and CHH sites under different sequencing depths and distributions of MeDIP-Seq reads in different CG density regions [20].

Methylation peaks (known as genome-wide highly methylated regions: regions with more than 20 sequencing tags and *p* value < 1 × 10^−5^) were scanned using MACS 1.4.0 (http://liulab.dfci.harvard.edu/MACS/, accessed on 10 May 2022). We analyzed the distribution of peaks in different components of the sheep genome (upstream2k, 5′UTR, CDS, Intron, 3′UTR and downstream2k). To identify the differentially-methylated peaks between Han ++ and HanBB groups, we assembled peak regions and calculated the number of reads of each sample. The numbers of reads in each peak were assessed using chi-square. False positive data were prevented through the false discovery rate (FDR) statistical method, significant at *p* ≤ 0.01. Only those peaks with read coverage from two groups of identical genomic components and significant differences of more than two-fold in reads between the two groups were identified as differentially-methylated peaks. All genes within differentially-methylated peaks were annotated using gene ontology (GO) analysis and Kyoto Encyclopedia of Genes and Genomes (KEGG) pathway analysis. The terms and pathways were significant at FDR < 0.05 [20]. This was followed by a sequence alignment and data analysis, referring to the original method, followed by tagging.

### 2.5. Co-Analysis of Differentially Expressed and Methylated Genes

The differentially-expressed genes between ovaries of Han BB sheep were found in our previous RNA-seq study [13]. In this paper, we studied differences in the methylation peak distribution of these differentially-expressed genes and identified genes with mRNA levels and DNA methylation differences between Han BB ewes.

### 2.6. Bisulfite Sequencing

For MeDIP-seq data validation, we selected specific gene PAGE4 and ADAT2 for bisulfite sequencing. Individual DNA samples in each group were treated with the EZDNA Methylation Gold Kit 500 µg, followed by incubation at 95 °C for 10 min and then at 64 °C for 2.5 h for bisulfite conversion. Then, we analyzed the modified DNA samples by bisulfite sequencing PCR. Bisulfite sequencing primers are listed in Table 1. The PCR was performed in a 25 μL reaction mixtures that contained 2 μL modified DNA template, 1μL primers for forward and reverse strand each, 0.15 μL Taq enzyme, 2.5 μL 10× PCR buffer, 1 μL Mg^2+^ (50 mM), 0.5μL dNTP (10 mM) and 17.85 μL ddH2O, with the following program: 94 °C for 2 min, followed by 10 cycles of denaturation at 94 °C for 30 s, annealing at 60 °C to 50 °C Δ1 °C temperature ladder for 30 s, extension at 72 °C for 30 s, and then followed by 30 cycles of 94 °C for 30 s, 50 °C for 30 s, 72 °C for 30 s, with a final extension at 72° C for 5 min. We purified and subcloned PCR products into pEASY—T1 vectors (transgen) for sequencing.

### 2.7. Statistical Analysis 

All data were processed by SPSS22.0 software and GraphPad Prism Version 7.0. The continuous variables were shown as mean (x ± s). We determined significant differences in data between the groups through one-way ANOVA followed by the Student’s *t*-test. One-way ANOVA and factorial design ANOVA were used for comparison of more than two groups. The test level was α = 0.05, and significant at *p* < 0.05.

## 3. Results

### 3.1. Global Patterns of DNA Methylation in Han ++ and HanBB Sheep

We mapped the global DNA methylation status of ovarian genome in Han ++ and HanBB sheep using MeDIP-seq. Ovarian tissue samples from Small-Tail Han sheep with wildtype BMPR-1B or fecB mutant were collected to generate the pooled DNA samples Han ++ and HanBB, respectively. A total of 5.55 Gb data within 113,264,664 raw reads were obtained from Han ++ sample, and 5.63 Gb data within 114,963,972 raw reads were obtained from Han BB sample. In two groups, 106,503,861 (94.03%) reads in Han ++ and 107,924,232 (93.88%) reads in HanBB sheep were successfully mapped to the sheep reference genome (ftp://ftp.ncbi.nih.gov/genomes/Ovis_aries, accessed on 10 May 2022), respectively. 63,333,133 reads (Han ++) and 64,171,269 (HanBB), respectively, were uniquely mapped.

**Figure 1:**
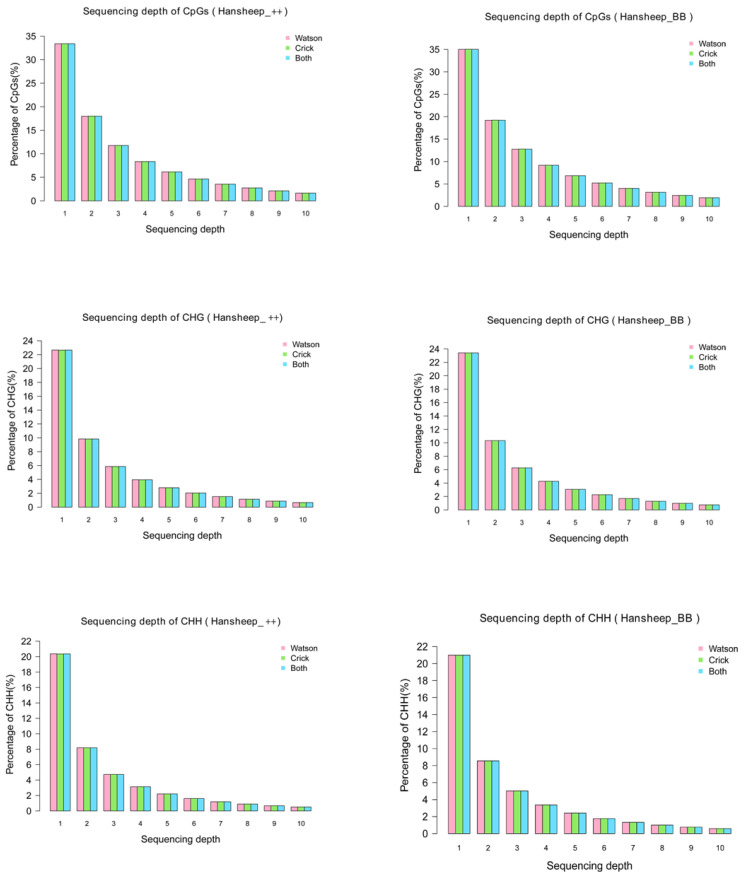
Sequencing depth of CpG, CHG and CHH in Han ++ (up) and HanBB (down) sheep. The percentages of the sequencing coverage of CpG, CHG and CHH in Han ++ sheep genome are shown in red (Watson strand), green (Crick strand) and blue (Both strand), respectively.

The distribution of unique mapped reads was detected in GGA1-26 and chromosome X (Figure S1), indicating that the MeDIP-seq covered all chromosomes of two kinds of Han ewes. Most cytosine methylation presents at the CpG dinucleotides. Some studies have shown that DNA methylation can occur at the C residue of some other sites, such as CHG and CHH (H refers to A, C, or T) [22]. In this paper, nucleotide sequences, rich in methylated cytosine at both CpG and non-CpG (CHG and CHH) sites, were precipitated and analyzed. The genome coverage of the CpG and non-CpG sites indicated that no more than 35% of CpG sites, and no more than 21% of CHG and 23% of CHH sites, had only one sequencing depth in two Han sheep breeds (Figure 1).

The distribution of mapped reads in genomic function regions of Han ++ and HanBB sheep was further examined. In both sheep, the methylated reads were mainly distributed at the gene body regions, CpG islands and introns. Normalized depth analysis illustrated that highly-methylated reads were present at the intragenic regions, but not the upstream or downstream 2 K areas (Figure S2).

### 3.2. DNA Methylation Peaks in Han ++ and HanBB Genome

**Figure 2:**
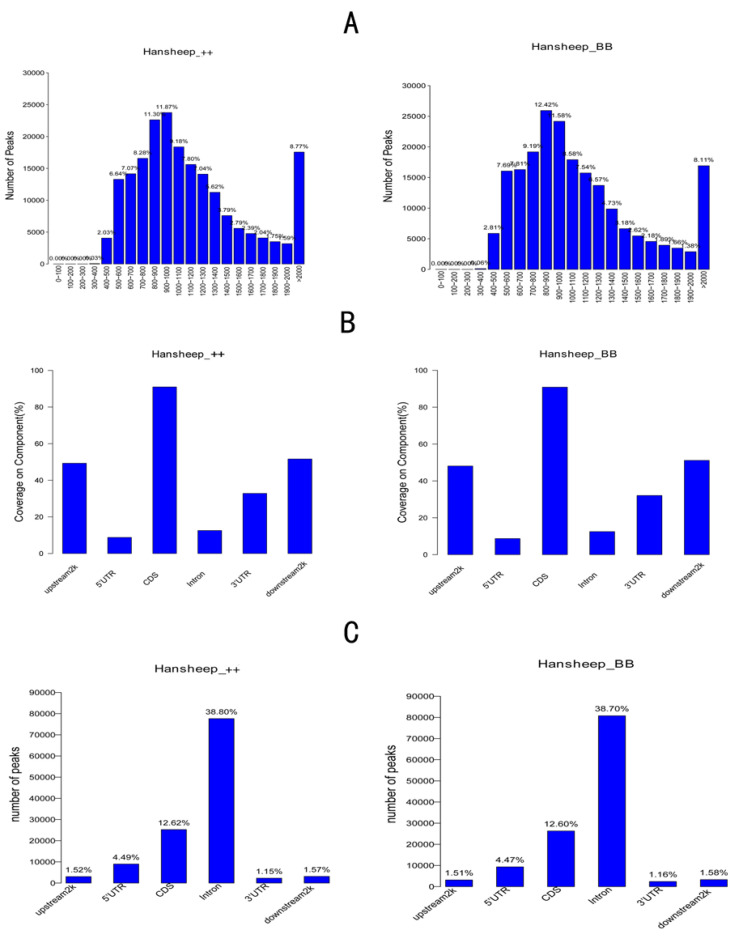
Methylated peaks’ distribution in different gene elements. (**A**) The number of methylated peaks in different length; (**B**) the coverage of methylated peaks in the intragenic (5′UTR, CDS, intron, 3′UTR), upstream 2 kb or downstream 2 kb regions; (**C**) the number of methylated peaks distributed in the intragenic (5′UTR, CDS, intron, 3′UTR), upstream 2 kb or downstream 2 kb regions.

To study the differences in genomic methylation patterns between Han ++ and HanBB sheep, we detected the 5-mC enriched regions, termed methylated peaks, by MACS1.4.0. A total of 200,158 peaks with coverages of 8.94% were identified. The typical peak lengths were 500–1400 bp or more than 2000 bp. Nearly one-fourth of peaks contained 10–15 CpG dinucleotides, and less than 20% of peaks contained more than 35 CpG sites (Figure 2). In two types of sheep, methylation peaks covered 90% of CDS, and 50% of upstream2k and downstream2k components were methylated. In the two types of sheep, the number of peaks in the intron region accounted for nearly 39% of the total peaks, and the coverage of peaks in the intron region was 12.5% in whole genome-wide (Figure 2).

### 3.3. Differentially Methylated Genes between Han ++ and Han BB Ewe’s Ovaries

After analyzing the DNA methylation patterns in Han ++ ewe ovaries using MeDIP-seq, we studied the differences in methylated genes, defined herein as genes with methylation peaks in intragenic regions between Han ++ and HanBB sheep, to investigate the effects of the FecB gene on ovarian genome methylation (Table S1). In HanBB ewes’ ovarian genome, in the upstream2k, 5′-UTR, CDS, intron, 3′-UTR and downstream2k elements, 382, 461, 1329, 3470, 249 and 114 genes, respectively, were at lower methylated levels than that in Han ++ sheep. However, 105, 275, 472, 2485, 32 and 91 genes in HanBB sheep were at higher methylated levels than that in Han ++ sheep, respectively. In general, the methylated levels of genes in HanBB sheep were lower than those of Han ++ sheep (Figure S3).

### 3.4. GO Annotation of Differentially Methylated Genes

The roles of differentially-methylated genes in biological processes between HanBB and Han ++ sheep were investigated using GO annotation (Table S2). Compared with Han ++ sheep, the genes with down-methylated upstream2k in HanBB ewes’ ovaries were significantly enriched in protein modification by small protein removal and innate immune response activating cell surface receptor signaling pathways. Genes with down-methylated CDS were enriched in a series of neuron development-associated functions, such as regulation of dendrite morphogenesis, regulation of neuron differentiation and generation of neurons. In addition, several processes, including cell adhesion, somatic diversification of T cell receptor genes, negative regulation of striated muscle tissue development and symbiosis, encompassing mutualism through parasitism, were enriched. Genes with lower methylated levels of intron elements in HanBB sheep were enriched in locomotion, cellular protein modification process, Calcium ion transport, G-protein coupled receptor signaling pathway, neuron projection morphogenesis and response to external stimuli. In the downstream2k region, down-methylated genes in HanBB sheep were enriched in synapse assembly, endoplasmic reticulum Calcium ion homeostasis, cell cycle G2 phase, glutamate receptor signaling pathway and mRNA 3′-end processing. However, those genes with down-methylated 5′-UTR or 3′-UTR were not significantly enriched in GO annotation.

On the other hand, genes with up-methylated upstream2k in HanBB ewes were significantly enriched in urea transport. Genes with up-methylated CDS were enriched in regulation of histone modification, DNA methylation on cytosine, modulation by virus of host cellular process and negative regulation of glial cell differentiation. Genes with up-methylated intron were enriched in microspike assembly, nucleotide catabolic process, regulation of cell communication, protein phosphorylation, regulation of signaling and metal ion transport. Genes with up-methylated 5′-UTR, 3′-UTR and downstream2k had no enrichment. In general, FecB gene-connected DNA methylation variations affected biological processes such as neuron development, Calcium ion transport, G-protein coupled receptor signaling pathway, histone modification and DNA methylation in Small-Tail Han ewes’ ovaries.

### 3.5. KEGG Pathway Analysis of Differentially Methylated Genes

The KEGG pathway database was used to study the pathways associated with differentially-methylated genes (Table S3). Compared with Han ++ sheep, genes with lower methylated levels of CDS elements in HanBB sheep were enriched in dilated cardiomyopathy, ECM-receptor interaction, neurotrophin signaling pathway, pentose phosphate pathway, GnRH signaling pathway, Calcium signaling pathway and tight junction. In particular, genes involved in GnRH signaling pathway, such as GnRHR, CACN, CaMK and Elk1, directly contributed to hyperprolificacy in HanBB sheep. Genes with down-methylated intron were enriched in axon guidance, ErbB signaling pathway, Calcium signaling pathway, dilated cardiomyopathy, tight junction and GnRH signaling pathway. Interestingly, genes with down-methylated intron in GnRH signaling pathway included CACN, Sos-Ras-Raf1-Elk1 and IP3R-CaM-CaMK. In addition, genes with down-methylated upstream2k element were enriched in neurotrophin signaling pathway, spliceosome, phenylalanine metabolism, arginine and proline metabolism, Wnt signaling pathway and oocyte meiosis. Identified genes involved in the oocyte meiosis pathway included Rsk1/2, Sgo, PP2A and PP1. Genes with down-methylated downstream2k were enriched in aldosterone-regulated sodium reabsorption, citrate cycle, vitamin digestion and absorption and glycine, serine and threonine metabolism.

Compared with Han ++ sheep, those genes in HanBB ewes with higher methylated levels of CDS were mainly enriched in ECM-receptor interaction, cysteine and methionine metabolism, pathways in cancer, gap junction, purine metabolism, arginine and proline metabolism, Calcium signaling pathway and GnRH signaling pathway. Those identified genes in GnRH signaling pathway included EGFR, MEKK and PLD. Genes with up-methylated intron were enriched in long-term potentiation, Calcium signaling pathway, GnRH signaling pathway, ErbB signaling pathway, phosphatidylinositol signaling system, Wnt signaling pathway, etc. In particular, genes involved in GnRH signaling pathway included AC, PKA, PLCbeta, PLA2, PLD and JNK. Genes with up-methylated downstream2k were enriched in vasopressin-regulated water reabsorption, natural killer cell mediated cytotoxicity, Toll-like receptor signaling pathway, T cell receptor signaling pathway, carbohydrate digestion and absorption, VEGF signaling pathway and mTOR signaling pathway. Those genes with up-methylated 5′UTR, 3′-UTR and upstream2k were not significantly enriched.

### 3.6. MeDIP-seq Data Validation

The DMRs identified from MeDIP-seq were validated through the bisulfite sequencing strategy. The bisulfite sequencing results of PAGE4 and ADAT2 genes were in accordance with the MeDIP-seq results (Figure S4).

### 3.7. DNA Methylation and Gene Expression Co-Analysis

**Figure 3:**
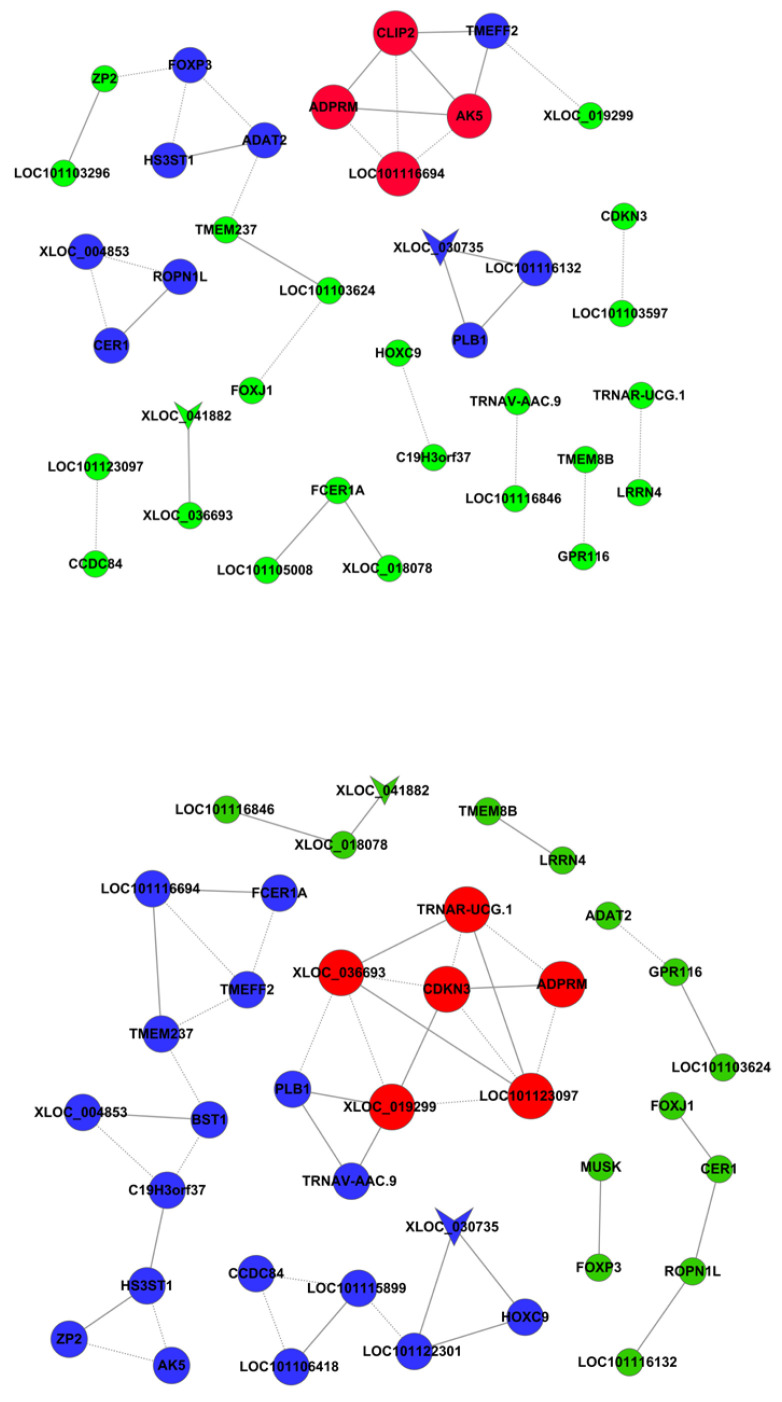
Co-expression network of differentially-expressed and -methylated genes. The vee nodes represent methylation genes. The circle nodes represent different genes. The solid lines indicate positive and the dotted lines indicate negative. The genes of the same color indicate the same co-expression. The co-expression of the same color gene is the same.

DNA methylation regulates gene expression. The differentially-methylated genes were co-analyzed with differentially-expressed mRNAs to study the roles of DNA methylation in FecB gene-related ovarian gene expression changes in Small-Tail Han sheep. The genes with different expression levels and methylation levels between HanBB and Han ++ sheep were AK5, PLB1, LOC101103624, GPR116, MUSK, LOC101103296, FOXP3, ADAT2 and TMEFF2. The co-expression network of differentially-expressed and -methylated genes is presented in Figure 3.

## 4. Discussion

DNA methylation refers to the process of adding a methyl group to the 5th atom in the pyrimidine ring of cytosine residues to form 5-methylcytosine. This mainly occurs in the cytosine-phosphoguanine (CpG) dinucleotide, catalyzed by DNA methyltransferases. The major methyl donor is S-Adenosyl methionine [23]. DNA methylation is involved in many important biological processes associated with chromatin regulation, including heterochromatin formation, centromeric and repetitive elements silencing and X-chromosome inactivation [24].

Additionally, DNA methylation can express genes involved in gametogenesis and embryogenesis [25,26]. In sheep, DNA methylation contributes to the characteristics of fertility [27]. However, it is unclear whether the FecB gene is responsible for these differences. To study the roles of the FecB mutant in DNA methylation changes, we examined the distribution of 5′-mC-rich regions on the ovarian chromatin of Han ++ ewes through MeDIP-seq strategy, and then compared the DNA methylation patterns of Han BB and Han ++ sheep.

Our methylomic study indicated that the methylated reads from Han ++ ewes’ ovarian genomes were mainly distributed at the intragenic regions, rather than the upstream or downstream 2 K areas. DNA methylation regulates gene expression depending on the methylated sites. Methylation, in promoters or body genes, is usually associated with gene repression or activation [28]. The hyper-methylated reads were enriched in intragenic regions. This indicated that the ovarian gene expression could be regulated via DNA methylation in Han ++ ewes. In addition, the analysis of methylated peaks indicated that highly methylated elements were enriched in CDS, rather than introns. This indicated that the coding exons were hypermethylated, whereas the introns were hypomethylated. The methylation level of promoter regions (upstream2k and downstream2k) was determined on a case-by-case basis.

We detected the differentially-methylated genes in order to identify genes affected by DNA methylation. The functional annotation indicated that genes in the GnRH signaling pathway, such as GnRHR, CACN, CaMK, Elk1, AC, MEKK, PKA, PLCbeta, PLA2, PLD and JNK, were identified in either down-methylated or up-methylated intragenic regions. GnRH signaling pathway played the central role in female reproduction [29,30]. Some studies showed that FecB stimulated fecundity via GnRH/LH/FSH endocrine axis [31,32]. A recent study reported that DNA methylation differences were identified in GnRH signaling pathway between endometrium and endometriotic tissues [33]. The results strongly indicated that FecB gene could lead to different DNA methylation patterns that play a role in GnRH pathway.

DNA methylation plays an important role in gene expression modification. Therefore, we applied co-analysis to the MeDIP-seq data and our previous RNA-seq data. Unfortunately, only nine differentially-expressed genes were identified with DMRs. Among these, compared with Han ++ sheep, FOXP3 had reduced DNA methylation levels in both CDS and intron regions in HanBB sheep. The expression levels of FOXP3 in HanBB were lower than in Han ++. This indicated that the DNA methylation difference between HanBB and Han ++ could lead to the expression difference of FOXP3. FOXP3 is a key transcription factor involved in immune system function, particularly in the development and function of regulatory T cells (Treg) in mammals such as sheep [34]. The difference in the expression and DNA methylation levels of FOXP3 between HanBB and Han ++ could be associated with the immune functions of the ovary. It will be necessary to study roles of Treg in ewe reproduction. In addition to FOXP3, TMEFF2 and ADAT2 may be associated with female reproduction. TMEFF2 is a transmembrane protein within two follistatin-like domains [35]. Existing studies have shown that TMEFF2 has the ability to inhibit the corticotropin-releasing hormone (CRH) signal pathway [36]. CRH was involved in ovarian steroidogenesis and suppress oocyte maturation [37,38]. This indicated that TMEFF2 could play regulatory roles in ovarian function. The methylation of TMEFF2 was identified in tissues from lung cancer patients, associated with reduced TMEFF2 expression level [39]. This indicated that the expression of TMEFF2 could be epigenetically regulated. However, the roles of TMEFF2 in ewe reproduction remain unknown, and require further study. ADAT2 is a subunit of tRNA-specific adenosine deaminase that catalyzes the formation of inosine at the first position of the anticodon [40]. Mutations in tRNA synthesis-related genes may disrupt oogenesis and lead to female sterility [41]. Could ADAT2 play a similar role in fecundity? The lack of investigation into these genes and their functions in female reproduction limited understanding of our results.

## 5. Conclusions

In this paper, we constructed a MeDIP-seq-based methylomic study to investigate differences in ovarian DNA methylation between Small-Tail Han sheep with homozygous FecB mutant and wildtype. We successfully identified FecB gene-related differentially-methylated genes. There have been few—and very limited—studies on the effects of a certain genes’ single-nucleotide polymorphism on genomic DNA methylation variations. This study provided novel information on the mechanisms of FecB gene-induced sheep hyperprolificacy.

## Figures and Tables

**Table 1:** Primers used for bisulfite sequencing PCR.

gene_id	Forward Primer	Reverse Primer
PAGE4	AGTAGGAATAGTGGGTTGTTGT	CACAAACTTAATATATCAATAAACT
ADAT2	GAAGGGATGATGGTTTATTAGGA	CGAACTATCTCCCTCAACATCAA

## Data Availability

The accession number GSE107829 for DNA-Seq data. https://www.ncbi.nlm.nih.gov/geo/query/acc.cgi?acc=GSE107829. The accession number GSE107935 for RNA-Seq data. https://www.ncbi.nlm.nih.gov/geo/query/acc.cgi?acc=GSE107935. accessed on 10 May 2022.

## Supplementary Materials

The following supporting information can be downloaded at: https://www.mdpi.com/article/10.3390/genes14010203/s1. Table S1: Methylation_HanBBvsHan ++_different. Table S2: Methylation_HanBBvsHan ++_GO analysis_result. Table S3: Methylation_HanBBvsHan ++_PathWay analysis_result. Figure S1: Reads distribution on chromosomes in Han ++ sheep (up) and HanBB sheep (down). Figure S2: Methylated reads distributionThe proportion of methylated reads in the eight elements of genome of Han ++ and HanBB sheep. Figure S3: Differentially methylated genes at different locations in the genome; Figure S4: Validation of RNA-seq and MeDIP-seq.
