# Supplementary figures and images for: Impact of FecB Mutation on Ovarian DNA Methylome in Small-Tail Han Sheep
Source: Genes (Basel). 2023 Jan 12;14(1):203. doi: 10.3390/genes14010203 (PMC9859159; doi:10.3390/genes14010203)

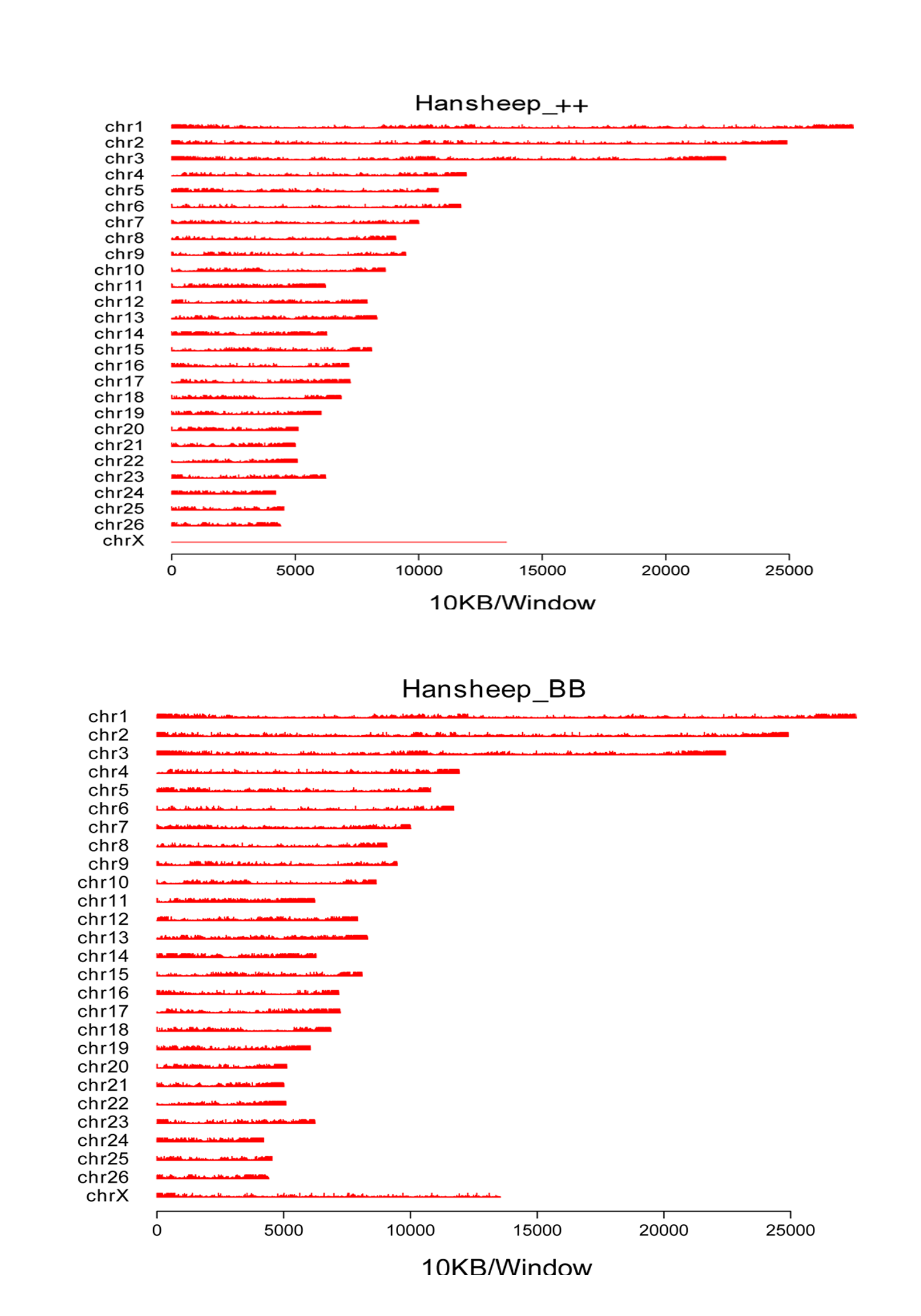

Supplement: Supplementary file 1 [file genes-14-00203-s001.zip › genes-2075644-supplementary/Figure 1S Reads distribution on chromosomes600_600dpi.tif]

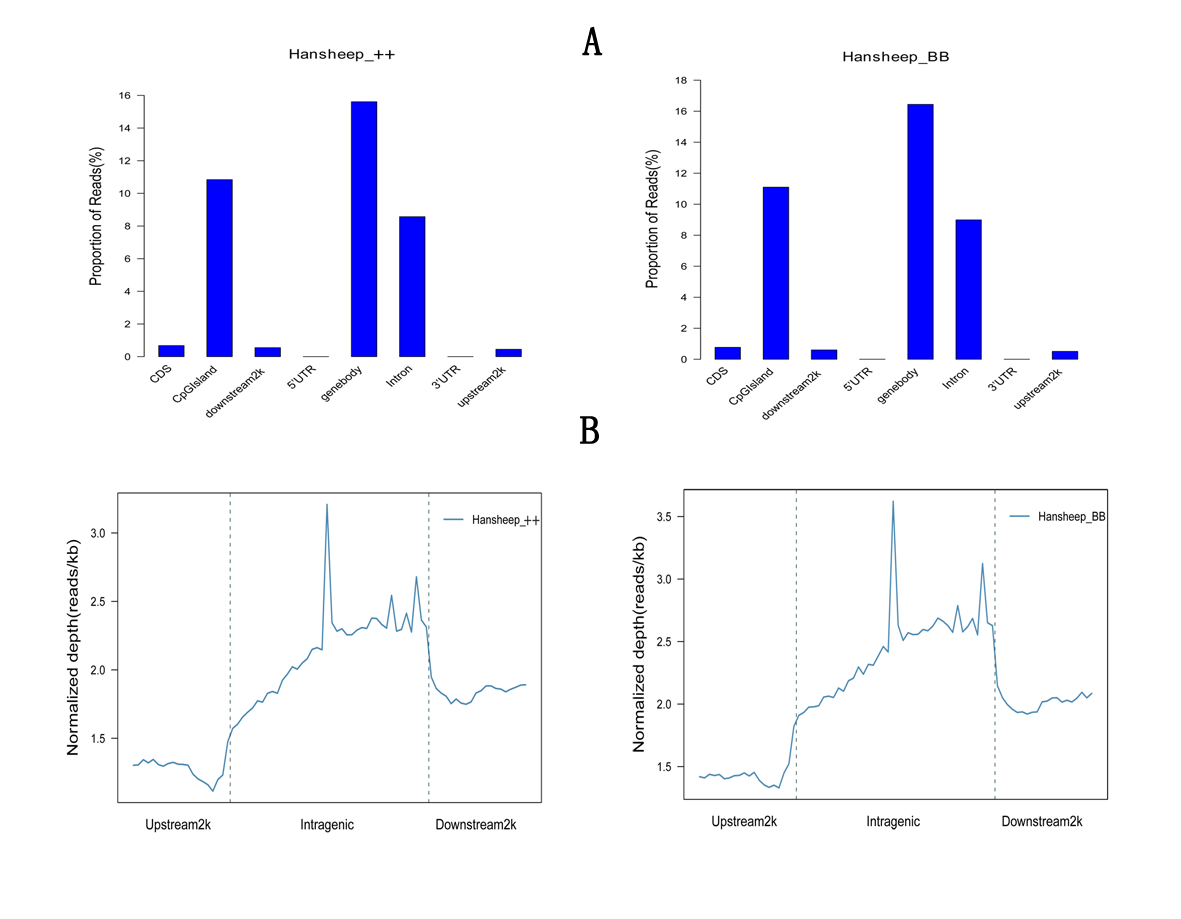

Supplement: Supplementary file 1 [file genes-14-00203-s001.zip › genes-2075644-supplementary/Figure 2S Methylated reads distribution._600dpi.tif]

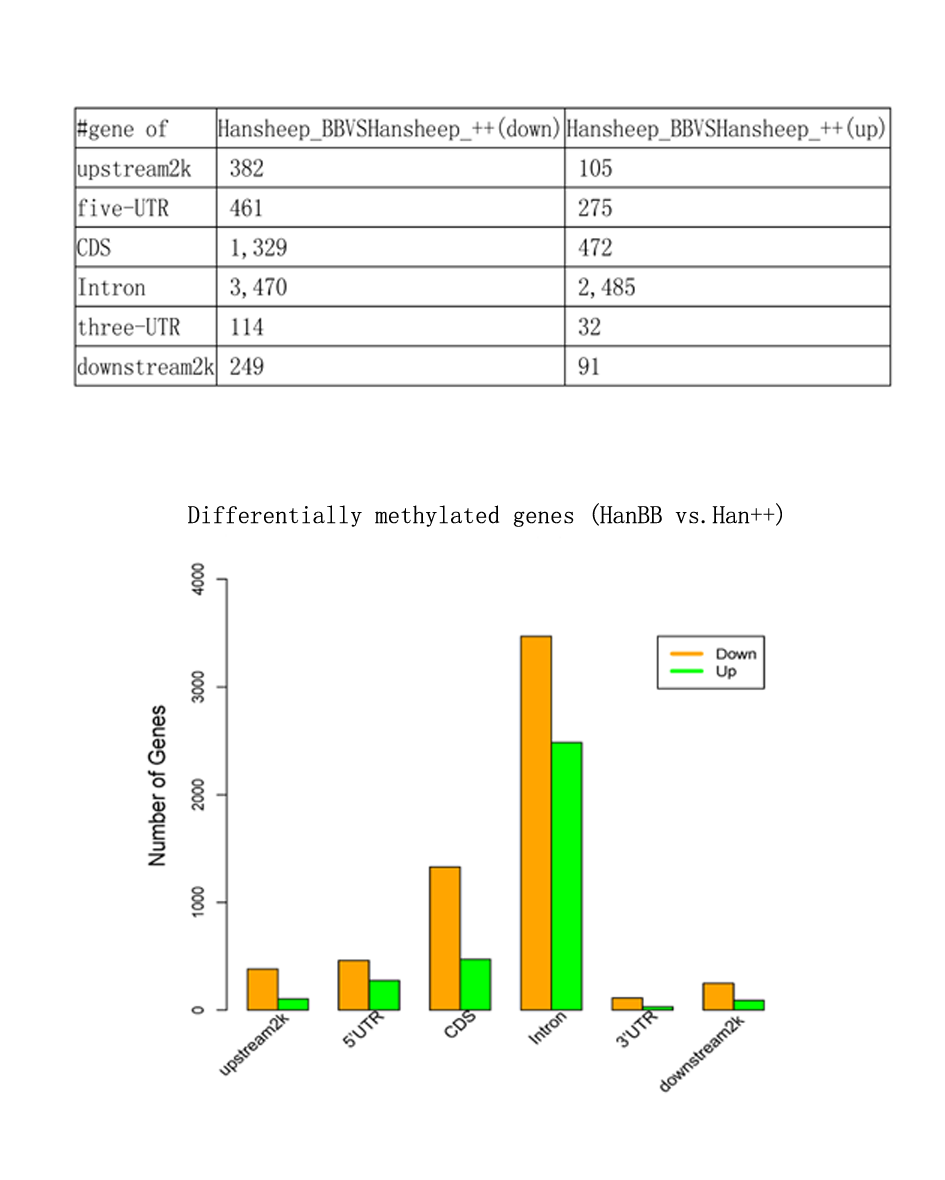

Supplement: Supplementary file 1 [file genes-14-00203-s001.zip › genes-2075644-supplementary/Figure 3S The difference of methylated genes_600dpi.tif]

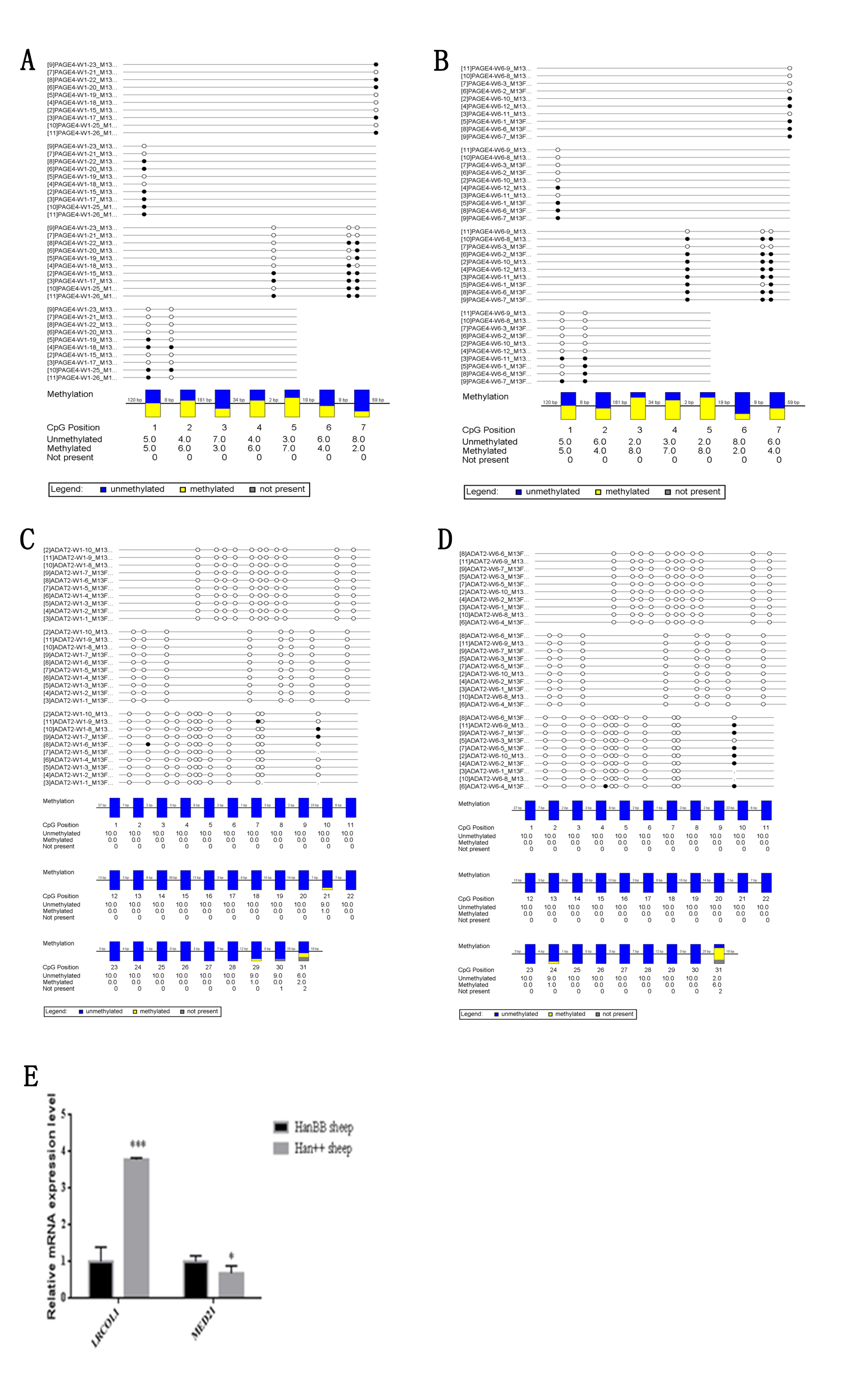

Supplement: Supplementary file 1 [file genes-14-00203-s001.zip › genes-2075644-supplementary/Figure 4S validation of RNA-seq and MeDIP-seq_600dpi.tif]
